# Supplementary material for: Dynamic Regulation of Mitochondrial [Ca2+] in Hippocampal Neurons
Source: Int J Mol Sci. 2022 Oct 14;23(20):12321. doi: 10.3390/ijms232012321 (PMC9604040; doi:10.3390/ijms232012321)
Supplement: Supplementary file 1 [file ijms-23-12321-s001.zip › ijms-1858074-supplementary.pdf]

Supplementary Materials

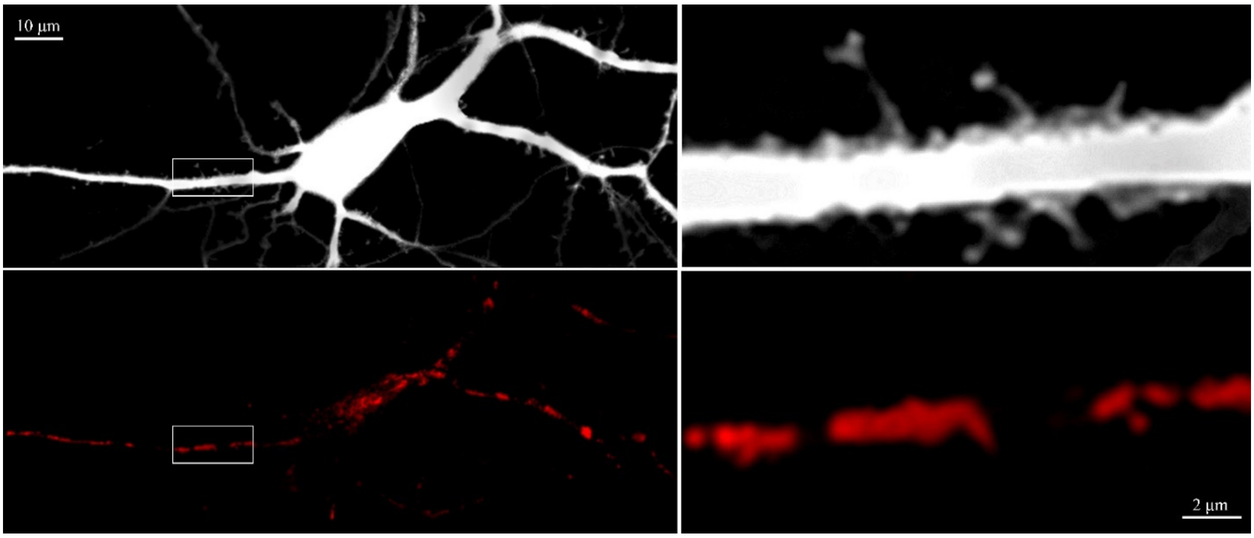

Figure S1. Low power (**left**) and high power (**right**) images of the neuron, visualized before the experimental recording from an area, shown in Figure 1A. **Top, left.** Overall cell morphology, BFP. **Top right**. Enlarged box area from the left panel. **Bottom left and right**. Same for mtRCaMP. Images the two sensors were taken separately to avoid crosstalks.

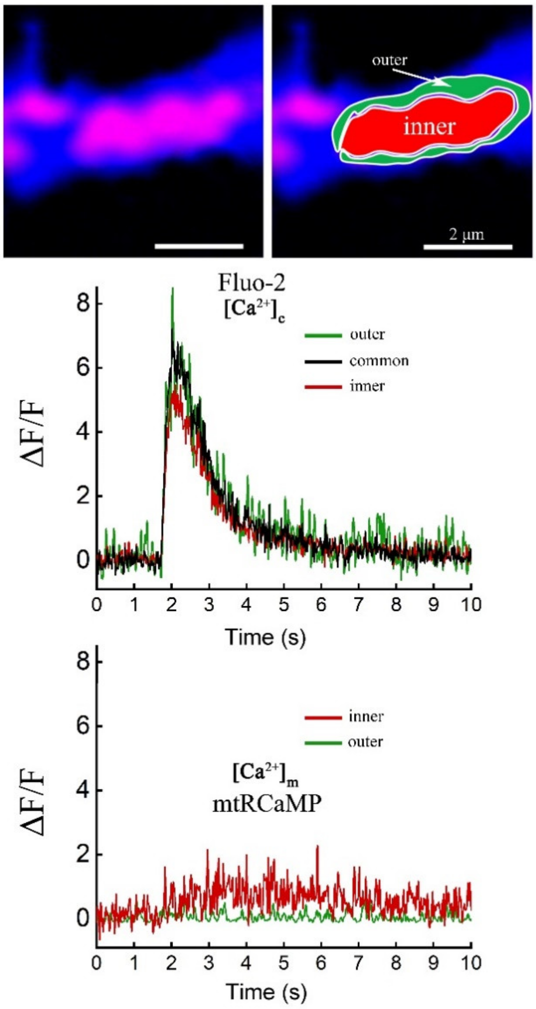

Figure S2. Fluo-2 and mtRCaMP calcium sensors represent calcium signals in different cell compartments. **Top**, **left.** Example of a typical dendritic mitochondrial cluster. **Top, right.** Sharp mitochondria contour (inner area, red), the surrounded cytosol (outer area, green) and the sum of the

two representing regions of calcium measurements. **Upper plot.** Fluo-2 traces for inner (red) outer (green) and common (black) areas, taken during cytosolic calcium event. Note slightly higher amplitude and increased noise in the outer compared to inner area due to their location and different surface. **Lower plot.** mtRCaMP traces in the inner (red) and outer (green) areas during the same event. Note lack of calcium signal in the outer area.

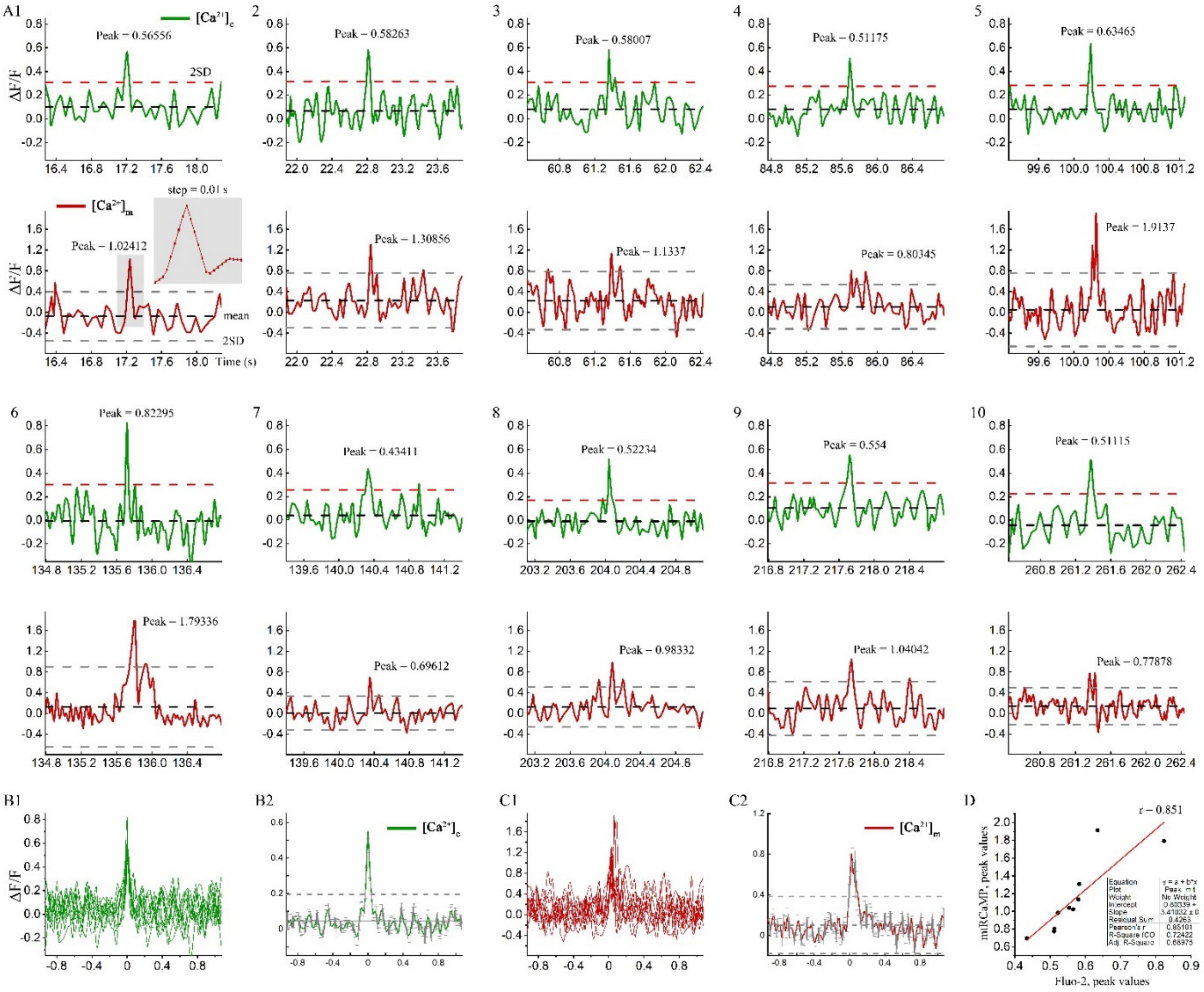

Figure S3. Single trace data set from Figure 1, (B1) the middle region. (**A1–A10**). Fluo-2 (cytosolic calcium, [Ca^{2+}]c, green) and mtRCaMP (mitochondrial calcium, [Ca^{2+}]m, red), 2 sec long traces, containig a sequence of 10 local calcium events, extracted from continues recording from the 'middle' (M) area of Figure 1, (**A&B1**). Only traces exceeding two standard deviation (2SD) criteria regarding the mean (dashed lines) have been considered. Insert in A1 (gray area) represents the enlarged mtRCaMP response with specific data points marked with red circles. Time resolution for all traces is 10 ms. (**B1**). All ten Fluo-2 traces overlayed. Time point 0 corresponds to the peak response. (**B2**). The averaged Fluo-2 trace, as in Figure 1, (**B1**), 'middle'. Single data points are provided with appropriate standard errors. Dashed line represents the 2SD for all averaged data points. (**C1,C2**). mtRCaMP traces, organized as in the panels **(B1,B2**). (**D**). Fluo-2 peak values (x axis), plotted versus mtRCaMP peak values (y axis) from (A1–A10) traces. Red line—linear correlation fit. Pearson's correlation coefficient (r=0.851) was calculated on the base of the peak values using OriginPro sowtware (lower right).

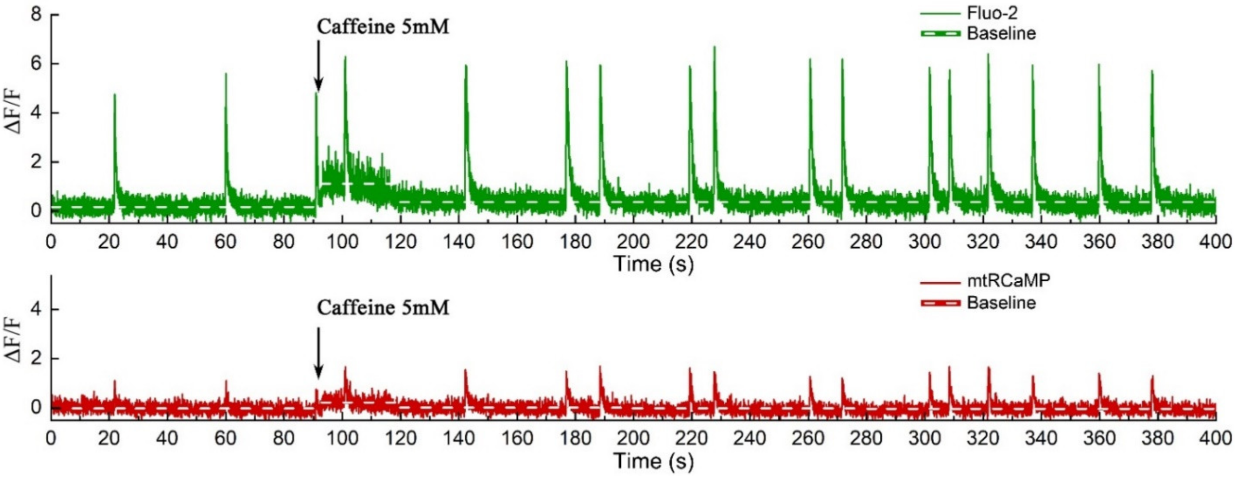

Figure S4. Effect of caffeine (5 mM) in soma. After the addition of the caffeinated solution, the baseline of cytosolic and mitochondrial calcium rises significantly, followed by a partial decline, but the level of cytosolic calcium remains elevated, while the level of mitochondrial calcium gradually returns to baseline.

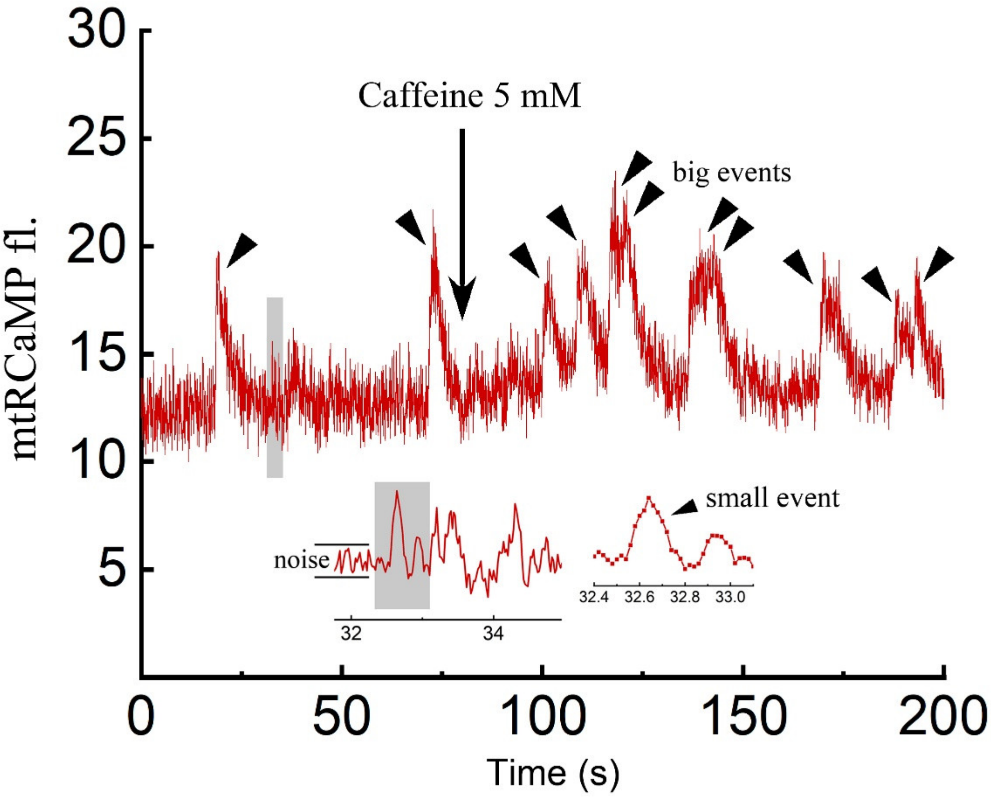

Figure S5. Effect of caffeine (5 mM) on mitochondrial calcium measured in the soma. The cell is transfected and imaged only with mtRCaMP. The addition of the caffeine causes an immediate rise in the baseline and frequency of events of mitochondrial calcium. Detected big events (possible global events) are marked with black arrows. An example of a small event (possible local event) in the gray area is enlarged in the insert panels.

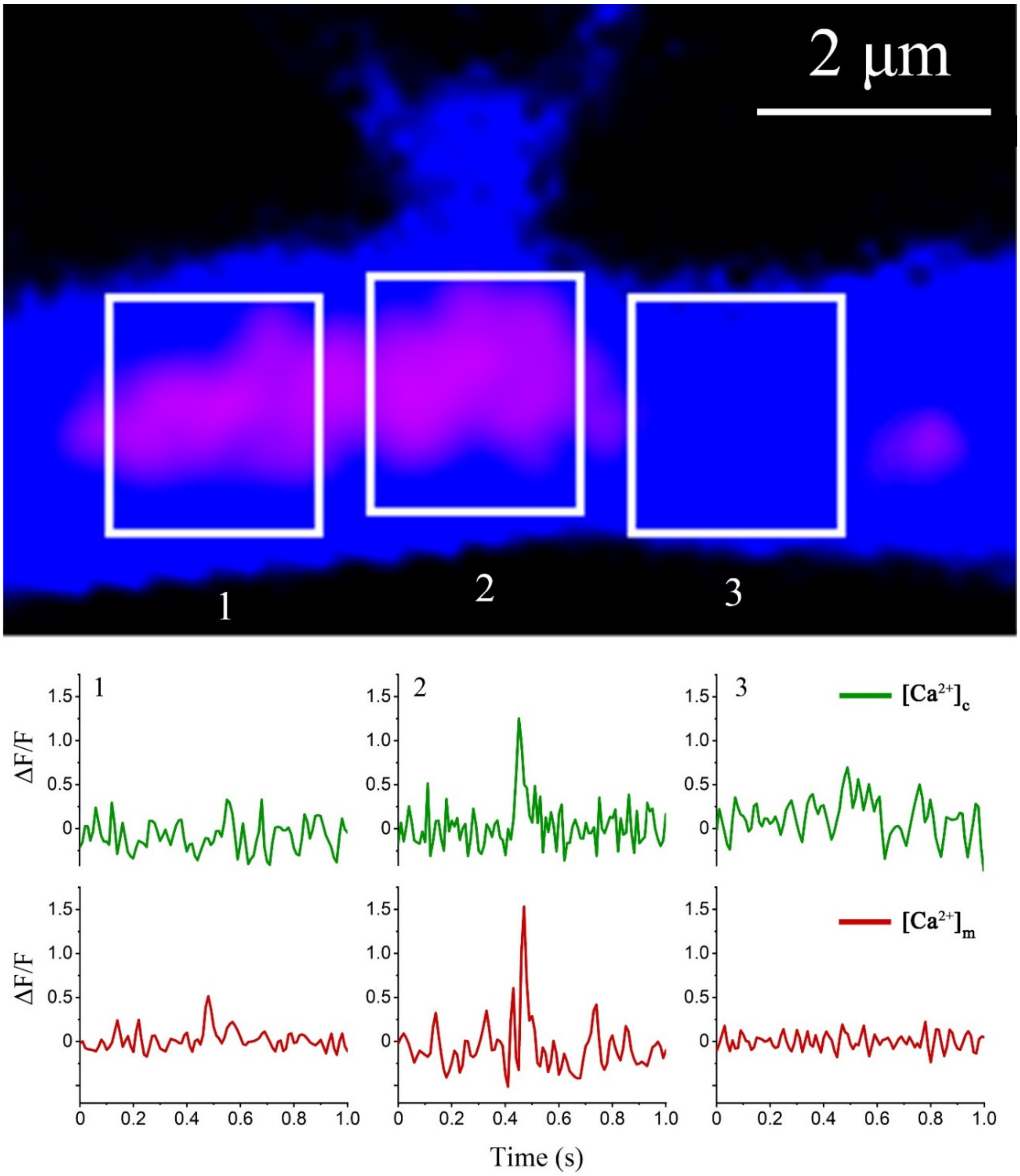

Figure S6. A local cytosolic calcium event is not fully absorbed in the absence of a mitochondrial cluster region. Region 2 contains a mitochondrial cluster that reacts with uptake for cytosolic calcium event, however, [Ca^{2+}]c event is able to propagate further down the dendrite to region 3 that does not contain mitochondria, as a result of which see the remainder of the cytosolic event in the signal, while in region 1, which also contains part of the cluster, there are practically no changes in [Ca^{2+}]c, only a slight increase in mitochondrial calcium is observed.
